# Philanthropic Motives of China’s Celebrities in Media Representation: From an Impression Management Perspective

**DOI:** 10.3389/fpsyg.2021.759671

**Published:** 2021-12-06

**Authors:** Jinghua Gao, Pengfei Zhang

**Affiliations:** ^1^Centre for Social Investment (CSI), Max-Weber-Institute for Sociology, Heidelberg University, Heidelberg, Germany; ^2^School of Labor and Human Resources, Renmin University of China, Beijing, China

**Keywords:** philanthropic motives, discourse analysis, self-presentation, impression management, celebrity philanthropy

## Abstract

**Background:** In China, celebrities, represented by entertainment and sports personalities, are often involved in charitable activities to assist the party-state in solving social problems. Although previous research has addressed the manifestation of prosocial behavior by Chinese celebrities, altruistic engagements have rarely been theorized from the perspective of impression management.

**Methods:** Based on the perspective of impression management, we use the discourse analysis approach to analyze the interview manuscripts of Chinese celebrities in media reports and then summarize the charitable motives and impression management strategies adopted by Chinese celebrities in their self-presentation.

**Results:** Chinese celebrities’ self-presentation of philanthropic motives in the media can be roughly divided into five categories: motivation for empathy-altruism, motivation for social responsibility, motivation to gain prestige, and pursue fame, motivation to reduce negative emotions, and motivation to achieve fulfillment and satisfaction. The philanthropic motives presented in media reports include the impression management processes of celebrities. They adopt a variety of image management strategies to self-present their philanthropic motives, and sometimes several strategies coexist.

**Conclusion:** Our paper helps to expand the existing understanding of the relationship between impression management and philanthropy. The presentation of Chinese celebrities’ philanthropic motivations in the media reflects the uniqueness of China’s political, institutional, and social environment in influencing celebrity philanthropy. As a philanthropic phenomenon with Chinese characteristics, this study could provide some insights into the understanding of celebrities and philanthropy in other cultural contexts.

## Introduction

Philanthropic motivation is the internal psychological process or motive that drives, directs, stimulates, and sustains an individual’s participation in charitable activities. Previous research has explored people’s philanthropic motivations and concluded that individuals’ willingness to give and volunteer is the result of a combination of factors. These factors include expectations of intrinsic benefits, such as increased self-esteem, satisfaction, and fulfillment (Dawson, 1988; Guy and Patton, 1989; Hibbert and Horne, 1996), as well as a sense of responsibility to help others in need (Hibbert and Horne, 1996), a desire to change society (Breeze and Morgan, 2009), religious concerns (Pharoah and Tanner, 1997; Schlegelmilch et al., 1997; Jackson, 2001), self-actualization (Sokolowski, 1996), and peer pressure (Andreoni et al., 1996; Glazer and Konrad, 1996).

In China, since the implementation of the reform and opening-up policy in the 1970s, political elites, economic elites, and public intellectuals have constituted the three main subjects of social participation (Levy and Pissler, 2020). Under the government’s efforts to combine politics and popular culture, celebrities represented by entertainment and sports stars often engage in charitable activities to assist the party-state in addressing national development issues (Deng and Jeffreys, 2019). They provide diverse forms of support to government-organized charities and government-endorsed international NGOs, which typically focus on poverty alleviation, disaster relief, child support and education, and environment and conservation issues (Deng and Jeffreys, 2019). Some celebrities serve on the boards of non-profit organizations and act as ambassadors for various charities and UN charity programs (Van den Bulck, 2018). Some celebrities are committed to public advocacy in the areas of environmental protection and human rights, drawing public attention to related issues through public service announcements and being an important part of achieving social and political change (Brockington and Henson, 2015). Some celebrities establish their own charitable foundations and social service organizations, taking the path of professional charity. For example, professional athletes, such as Yao Ming, have established foundations to demonstrate their prosocial behavior (Tainsky and Babiak, 2011). The emergence of the phenomenon of celebrity philanthropy is defined as a particular type of “soft activism,” in which celebrities participate through their charitable organizations (Palmer, 2021).

Although previous research has addressed the manifestations of prosocial behavior by Chinese celebrities (Jeffreys, 2015, 2018), altruistic engagements have rarely been theorized from the perspective of impression management. However, the existence of this phenomenon and mechanism reveals the strategic practice of celebrities using altruistic measures to construct, embody or set their own image. Although opportunities to create altruistic prosocial impressions in the media environment do exist (Duygu et al., 2019), little research has been conducted on the relationship between self-presentation and philanthropy. The bulk of the research has typically been conducted in controlled laboratory settings and with students as the primary participants (Ariely et al., 2009). Furthermore, the motivations of celebrities for self-presentation in the media can vary culturally, and we still know very little about self-presentation by celebrities in different cultures. In response to these gaps, this paper examines the practice and behavioral logic of celebrities’ image management by analyzing their attitudes and feelings of participation in charitable activities.

## Theoretical Perspective

Impression management is inseparable from self-presentation. According to the famous sociologist Goffman, society and life constitute a big stage for performance, and everyone is like a performer on the stage, attempting to portray themselves in front of the audience, with the aim of convincing the audience that they are a certain type of person, although they might not be (Goffman, 1949). To achieve the goal of persuading others to accept the image that the individual claims to have, Goffman suggested two types of self-presentation, namely acquisitive self-presentation, and protective self-presentation (Arkin, 1981). Both self-presentation behaviors are influenced by internal (personality) and external factors (audience characteristics) (Paulhus and Trapnell, 2008). The former attempts to render their positive efforts visible to others as much as possible, while the latter attempts to undermine their shortcomings or avoid having others view their defenses negatively. People always use symbols in daily life for expression to design or display the impression presented to others in advance. This process is defined as impression management, which is a goal-oriented activity of controlling information to influence the impressions formed by the audience, and it plays an important role in interpersonal interactions (Paulhus and Trapnell, 2008).

Studies have shown that celebrities focus on impression management to maintain or enhance their positive social image and ultimately gain more social and economic capital (Guo and Ren, 2020). The media, as an environment in which celebrities can freely present themselves, can largely exert a powerful influence on those who are closely associated with their media image (Fraser and Brown, 2002). Celebrities tend to actively present themselves in the online environment and achieve interactions with their fans (Marwick and Boyd, 2011). Celebrity discourse, especially media interviews, naturally constitutes a very common way for celebrities to present themselves.

Most celebrities employ impression management strategies when confronted with media interviews, with the aim of convincing their fans and the general public to accept or believe in the image that they portray. Celebrities’ fans and impersonators can develop strong identifications with them by consciously role modeling celebrities’ values and by changing their own lifestyles to emulate the celebrities (Fraser and Brown, 2002). In this way, the media acts as a showcase and becomes a stage for the self-presentation of entertainment and sports celebrities. The interview process can be regarded as a specific reaction of the celebrities according to a performance framework. Then, the words and pictures in the media serve as the set and props of the stage.

Self-presentation includes both impression motivation and impression construction (Duygu et al., 2019); celebrities have a desire to appear prosocial and altruistic, and they behave in certain ways to create this impression. Philanthropy, as an effective way to enhance a donor’s reputation for altruism or wealth (Harbaugh, 1998), is therefore widely accepted and applied by celebrities. The image motivation of “desire to be liked and respected by others” has received attention from researchers. Some studies have found that image motivation is a powerful tool for forcing prosocial behavior (Bator and Cialdini, 2000; Ariely et al., 2009). People show more generosity when they are observed or noticed in social settings (Reinstein and Riener, 2012). They are more inclined to be altruistic when seen by others for their good deeds, and public giving actually increases the probability of charitable behavior occurring (Andreoni and Petrie, 2004). Thus, those who want to be perceived as good people might act more generously or more socially.

Some celebrity donors might seek an experience of prestige when making their giving public, especially among those with higher social status and wealth (Ostrander, 1997). Among the various forms of self-presentation used by celebrities, positive charitable attitudes, and altruistic philanthropic behavior are positive statements about oneself that aim to construct a good self-image (Guo and Ren, 2020). To successfully demonstrate or prove their social responsibility, some celebrities like to use their front-page image as a means of self-grooming when they are interviewed by the media. For many of them, disseminating information about their charitable activities and charitable involvement in the media to fans and/or other audiences is not only a form of interaction but also a mode of self-presentation. Many celebrities use their words, pictures and videos to create and present their charitable images in front of the general public, with the aim of further transforming their popularity into commercial value or even covering up scandals.

## Materials and Methods

### Discourse Analysis

This study adopts the discourse analysis approach to analyze the interview manuscripts of Chinese celebrities in media reports and then summarizes the philanthropic motivations of Chinese celebrities presented in the media and to the public. Thus, the internal mechanism influencing the manifestation of these motivations is explored. Discourse analysis lies at the heart of a reconfigured psychology, which is one of the most widely used approaches within social constructionism (Jørgensen and Phillips, 2002). Discourse analysis is the study of how speech and texts are used to perform actions (Potter, 2003). The point here is that psychology is something alive and visible as it appears in and through discourse (Potter and Wetherell, 1987). Discourse analysts have access to a wide variety of data sources, including interview transcripts, focus groups, conversation samples, published literature, media, and web-based materials (Hodges et al., 2008).

In recent years, a growing number of discourse analysis studies have examined a variety of topics related to celebrities, particularly discourse studies on the management of celebrities’ relationships with their fans (Marwick and Boyd, 2011; Wu and Lin, 2017; Zhang and Wu, 2018). For example, celebrities connect with their fans by creating and sharing content, and they create a sense of intimacy between participants and followers (Marwick and Boyd, 2011). Some celebrities create a stance of misalignment that is inconsistent with mass media, using combative and corrective rhetorical strategies in interviews with journalists (Valentinsson, 2018). This stance suggests that celebrities can adopt different discursive strategies in the media, which is precisely why this paper use discourse analysis to study celebrities’ impression management of their philanthropic motives.

### Data Collection

The interview data for this article are drawn from mainstream media and magazines that cover the philanthropic activities of Chinese celebrities, including two major categories. The first category is media outlets dedicated to covering Chinese philanthropic activities. We focused on the *Public Interest Times* and *China Philanthropist* magazine. The former is China’s first comprehensive national philanthropy newspaper. The latter provides a comprehensive focus on philanthropy, wealth and social issues on a global scale. Therefore, we collected the texts of all celebrity interviews above in these two publications. The second category is celebrity coverage articles in mainstream media, covering Xinhua net, People’s Daily Online and China News Network.

To collect data that are more representative for this study, we focused on those who have been promoting philanthropy for a long time, who are engaged in specific philanthropic activities, and who have a certain degree of popularity and influence. Based on the China Philanthropists List published by *China Philanthropist* magazine and the Forbes China Celebrities List, the research samples selected consist of all Chinese celebrities with outstanding performance in the field of philanthropy and public welfare. The data were collected from 2013 to 2020. After removing similar and duplicate reports, a total of 134 media interviews were included in the analysis scope of this study.

### Data Analysis

Since this study aimed to shed light on a previously underresearched phenomenon, it is important to avoid imposing a predefined and inappropriate taxonomy (Guo and Ren, 2020). Therefore, within the framework of grounded theory (Charmaz, 2014), the 134 interview texts were coded with the help of NVivo (version 12). The analysis of the data proceeded as follows.

This study intends to interpret the collected celebrities’ philanthropic motivations in media coverage. We first read all of the interview texts and coded the categories of motivation according to their content. In the process of coding the data, it was found that some celebrities had more than one philanthropic motive. In this case, each individual motivation was coded separately. Second, after coding all of the interview texts, the main concepts and categories in the study were found, and similar philanthropic motivations were grouped into the same category according to the principles of exhaustion and mutual exclusion. Third, the categories of Chinese celebrities’ philanthropic motivations were abstracted and generalized according to the relationships between concepts and categories. The classification of philanthropic motivations comes from the existing literature, including empathy-altruism (Fultz et al., 1986; Batson, 1997; Cialdini et al., 1997), reciprocal altruism (Ramsey and Brandon, 2011), social responsibility motivation (Steele et al., 2008), reputation-seeking motivation (Reinstein and Riener, 2012), and reduction of negative emotions (such as guilt and anxiety) (Basil et al., 2008; Lwin and Phau, 2014).

Finally, to avoid subjective bias in coding, two coders were selected in this study as the main judge and comparison judge. Generally, the concept of reliability broadly describes the extent to which results are reproducible, for example, between two judges of behavior (Drost, 2011; McDonald et al., 2019). The coding results are considered acceptable when the agreement between the ratings of the main and comparison raters for the same code exceeds 80% (Landis and Koch, 1977). The interrater reliability in this study was 92.37%, indicating that the encoding reliability is reliable. For the problematic and controversial cases, the two coders discussed them and agreed after discussion.

## Results

### Motivation of Empathy-Altruism

The majority of celebrities employ idealized performance strategies to present an “ideal” self-image when faced with media interviews. Media interviews naturally become carefully rehearsed performances by celebrities and the media. Celebrities tend to portray themselves as caring people, suggesting that their charitable actions are driven by loving instincts. For example, many celebrities say that they are by nature like ordinary people, with instincts and a nature for love and goodness. Engaging in philanthropy stems from the love in their hearts, which is the fundamental source of motivation for their charitable acts.

*Philanthropy comes first and foremost from love. Love for people and everything around you are the sources of giving. (“Love for people and everything around you are the sources of giving,” 20170213TXW).*

Charity manifests the most essential value of human nature and embodies the pursuit of moral perfection. Many acts of altruism are based on compassion for others (Fultz et al., 1988). Some celebrities stated that they offer help to the poor and less fortunate out of compassion. Traits such as empathy, emotional vulnerability, and sympathy for the same and similar experiences often create an emotional connection between the celebrities and the recipients. The more compassionate that an entertainment celebrity is, the more likely that it is that he or she is involved in philanthropic endeavors. For example, one actress has persisted in making charity the driving force of her career for years precisely because of empathy.

*When I walked into the poorest mountainous area in Weining County, Guizhou Province, I was deeply stung by the living conditions of the children there, who were not well fed or well clothed. This made me very sad. (“Public figures should spread positive energy and lead to goodness,” 20150506RMW).*

*Five children suffocated to death in garbage cans at the same time. I am also a mother. At that time, I thought, in addition to shedding tears, shouldn’t I also help these children? (“Five Bijie boys suffocated in a garbage can made her fade out of the big screen,” 20160803TXW).*

Some celebrities, in contrast, first imagine themselves in the eyes of their fans and the public to understand how others perceive them and then reinforce their self-identity by playing the role of sympathizer in front of the media. This behavior is essentially self-image building and construction. For example, an actor was hired by China’s Ministry of Public Security as a “volunteer propagandist in the fight against abduction.” In a media interview, he said that his charitable act was motivated by emotional vulnerability.

*I am an emotionally fragile person. Whenever I see news of separated families and lost children and elderly people, my heart is especially hurt. (“Celebrities should do charity as they wish, don’t let the needy down,” 20180509FHW).*

### Motivation of Social Responsibility

As public figures and socially elite groups, Chinese celebrities have a sense of responsibility and mission and often feel obligated to give back to society. Not only do they want to set an example by giving to society, but also they want to act as spokespersons for public welfare, purify the social climate, and become role models for their audiences and fans. As a result, Chinese celebrities follow the strategy of “idealized performance.” They attempt to portray themselves as responsible public figures. Although some entertainment celebrities do not care about philanthropy, they use some of their rhetoric to depict responsibility as an important motivation for their charitable behavior.

*Celebrities and public figures should be the strongest advocates of the public good and charity. (“Celebrities and public figures should be more vocal for public welfare and charity,” 20190411ZGCSJ).*

*As a public figure, one should always be a good advocate with people. (“Hai Qing was awarded the title of ‘Goodwill Ambassador’ and spared no effort for charity,” 20110109SHW)*

*A star is first and foremost a citizen with his or her own due responsibilities and obligations. As a popular star, he or she should set an example and play an exemplary leading role. (“I’m often helpless as an actor, but I feel I’m useful for public welfare,” 20160505ZGZYZ).*

In contrast to idealized performances, celebrities sometimes portray themselves as mysterious by attending a large number of charity events to distance themselves from the public and demonstrate their ability to care about and support charitable causes. Examples include attending various charity dinners, participating in charity auctions, and serving as “charity ambassadors,” “ambassadors,” and “spokespersons for branded charity programs.”

*The influence of public figures is actually immeasurable… If public figures do something meaningful, the value of their influence is very high and can guide countless people. (“Public service is not only about giving, it is also a transfer of responsibility. Make good use of the influence of public figures,” 20190318ZGCSJ).*

Chinese celebrities are adept at using their influence to strengthen their advocacy for charitable causes, convey positive social energy and guide their fans to give generously. It has been the desire and insistence of many entertainment celebrities to support charitable causes and to fulfill their obligations. Celebrities advocate for public welfare, including promoting gender equality; fighting domestic violence; calling for wildlife protection; caring for refugees, women and children; helping the disabled; advocating for public welfare for all; fighting trafficking; promoting voluntary participation; eliminating discrimination against people with AIDS; etc.

*By setting up this fund, I hope to use the power of role models to get more people to participate in public welfare, rather than just asking for donations. (“Yi Yeoh has become the youngest cover character of China Philanthropist, topping the list of Chinese charity stars,” 20180208SHW).*

In addition, entertainment celebrities and sports celebrities also actively exert the power of role models to influence and drive more fans to promote the development of charity through their own participation. Celebrities, as “charity role models,” are inherently mysterious and can cause fans to imitate them and have a positive impact on youth.

*I want to influence my fans as much as I can, and then they can influence the people around them so I can spread the power of love. (“Ma Yili Shanghai sends ‘Little Love,’ attaches importance to cultivating her daughter’s love,” 20111110XLW).*

### Motivations to Gain Prestige and Pursue Fame

In reality, many celebrities are passionate public service to gain the respect of others or to be seen by others as good and honorable people. Therefore, they are more likely to show altruism in the public eye. Hopefully, the media play up their charitable acts and attempt to cause others to have a positive perception of their efforts and contributions to charity, thus believing that their good deeds are genuine.

*I hope I don’t make superficial statements and don’t rely on my status as an actor to be a good person but as an individual who also commands the respect of others. This has also been an intrinsic motivation to actively pursue philanthropy over the years. (“A Passage to Innovation,” 20150129ZGCSJ).*

*As for me personally, in a few years, I hope people will think I’m a good person, which is my expectation. (“As an actor, I’m often helpless, but I think I’m useful for the common good,”20160505ZGZYZ).*

In addition to gaining respect, celebrities use strategies of protective impression management to improve a bad image. We find that some celebrities do not truly care about philanthropy but use it as a tool to whitewash their transgressions and improve their bad public image. When their public image is damaged or their career is facing a crisis, they will undertake remedial measures to build a good social image to regain the attention of their fans and bring more benefits to their careers. This behavior is essentially a remedial performance.

For example, Fan Bingbing, a well-known Chinese entertainment star, suffered serious public image damage in 2018 due to a tax evasion incident. Since then, she has taken steps to return to the public eye by participating in charity events. In late 2019, she was awarded the honorary title of “Female Charity Ambassador” and received an annual charity award at the SOHU fashion show. These events subsequently drew serious public skepticism and criticism. Another entertainment celebrity shouted the slogan “Get out of the entertainment industry” during the low point of her career. At the most difficult time of her acting career, she used charity as a source of energy to improve her image and heal her wounds. This type of celebrity, who does not sincerely support charity but uses it as a tool and means to satisfy personal interests, has caused public outcry. It also constitutes the main reason why celebrities are suspected of having impure charitable motives.

In addition, there are celebrities who distort their image during disasters, portraying themselves as benevolent people when they are actually not willing to donate to charity. When the Wenchuan earthquake occurred in China, many celebrities promised to donate to support the disaster area, but some of them later did not fulfill their promises. This situation led to the exposure of donation fraud scandals. For example, the famous Chinese movie star Zhang Ziyi was deeply involved in a donation scandal that attracted widespread public attention (Jeffreys, 2015).

### Motivation to Reduce Negative Emotions

Previous research has shown that has shown that both sympathy and guilt enhance individuals’ prosocial behavior. Empathy and self-efficacy generate guilt and reduce maladaptive responses, in turn shaping donation intention (Basil et al., 2008). That is, the motivation to help, associated with empathic emotions, is directed toward the egoistical goal of negative state relief rather than the altruistic goal of relieving victims’ suffering (Batson et al., 1989). Although sympathy-induced altruism does increase helpfulness, it also reinforces the individual’s sense of sadness or guilt (Cialdini et al., 1987), leading to self-directed concerns and a desire to reduce the individual’s distress. The fundamental starting point of this egoistic motivation is to reduce one’s own discomfort. In other words, the effect of empathy on charitable intentions is mediated by guilt and adverse reactions. Guilt does increase an individual’s charitable intentions (Basil et al., 2008).

In contrast to ideal, misunderstood, and mysterious manifestations, instinctive manifestations are those in which celebrities are presented in media interviews largely consistently with how they themselves think and behave in reality. These interview articles were published without any retouching or processing. In other words, this self-presentation in the media coverage stage is highly coordinated with the real situation backstage, presenting the celebrity’s true self.

We find that some celebrity artists participate in charity events to reduce their discomfort, rather than out of positive emotions. For example, one celebrity presenter was so traumatized by her advocacy for animal welfare that she even needed to see a psychiatrist for professional treatment. However, she still decided to fight against humanity because she could not forget the tragic situation of these animals. She would feel guilty herself if she did not undertake concrete action.

*I wish I hadn’t seen this. If I didn’t know this, I would be much better off than I am now. But if I knew and didn’t care, I wouldn’t be able to stand it. (“Compassion for hearts,” 201508ZGCSJ).*

### Motivations to Achieve Fulfillment and Satisfaction

The deepest human desire or motivation is to find meaning and purpose in life. Chinese celebrities, like the general public, want to gain a sense of accomplishment, satisfaction and honor by helping others, which is not only an important experience for them to participate in public service but also an important motivation for them to persevere. Celebrities benefit themselves while helping others, such as by gaining recognition and moving others, purifying their souls, improving their character and mindset, feeling the joy of helping others, replenishing energy by doing good deeds, gaining healing, etc. These outcomes are the motivating sources for celebrities to engage in charitable activities.

For example, one actor said that, by doing good deeds, he develops sympathy and compassion for many things and people, and his personality gradually becomes peaceful. He becomes gentler and more comfortable when interacting and communicating with others.

*Many of my friends and I have become more compassionate about doing good deeds, and helping others gives us a greater sense of accomplishment. (“Interview with Basketball Superstar Yao Ming on Charity,” 20140718XHW).*

There are also celebrities who see philanthropy as a reciprocal act that not only benefits them but also supports a cause. Thus, a sense of accomplishment becomes one of the motivations for Chinese celebrities to be charitable, which is closer to their own true expression.

*I am not so much helping others as I am being helped by them. It is these touching moments in my life that purify my soul and fill my heart with love. (“Being able to help others is a blessing,” 20160524GYSB).*

*When I help others, they give me something spiritual in return, which makes me feel like I have done something meaningful. (“Charity ‘Addiction,”’ 20160415ZGCSJ).*

*Doing good is a process of “finding yourself, discovering more possibilities, knowing the meaning of love, knowing what your values are and what the meaning of life is.” (“Falling in Love with the Rural Children,” 20151209YGW).*

Other celebrities see caring, supporting and participating in social service work as important means of self-improvement, self-cultivation and self-redemption. They see prosocial behavior as a process of inner self-redemption, and they hope to achieve inner self-cultivation through charitable altruism, which is also a reflection of their true personality and character.

*The power to do good comes from an inwardly charitable heart. … The key to charity is to cultivate oneself. (“Doing Good, Acting to Make a Difference,” 20181204GYSB)*

*Doing good is a constant reminder to stand still and keep the truth, goodness and beauty in yourself. (“Huang Yi’s ‘Game Moves,”’ 20141223ZGCSJ).*

## Discussion

Based on the discourse analysis of Chinese celebrities’ media interviews, this study found that Chinese celebrities’ self-presentation of philanthropic motives in the media can be roughly divided into five categories: motivation of empathy-altruism (Batson et al., 2015), motivation of social responsibility (Khalifeh Soltani et al., 2021), motivation to gain prestige and pursue fame (Anderson, 2009), motivation to reduce negative emotions (Merchant et al., 2010), and motivation to achieve fulfillment and satisfaction (Mannino et al., 2011). The philanthropic motives presented in media reports include the impression management processes of celebrities. They either use idealized performances to present a charitable image of themselves to the media as the side that they are willing to show and want the public to see, which sometimes does not match or even has a very large gap with the image of their real selves; or they use a misunderstanding performance strategy, pretending that they are a generous and caring person and causing the public to believe that they are genuinely concerned about supporting charities. Either they use the mystification performance strategy to cause their fans to admire and imitate their charitable behavior or they use charity as a tool to salvage their public image and make a profit when their careers encounter setbacks and PR crises.

Celebrity philanthropy can take different forms and can be viewed differently in different parts of the world (Batson et al., 1989). In mainland China, it is a recent phenomenon that has attracted both media publicity and public controversy (Jeffreys, 2015). Celebrities use their public visibility, brand credibility, and personal wealth to promote not-for-profit organizations (Jeffreys and Allatson, 2015). Although China is the largest media market in the world, previous studies of celebrity philanthropy have been conducted almost exclusively in Western contexts (Hassid and Jeffreys, 2015). This study brings a new perspective to the discussion of celebrity impression management and philanthropy and provides the latest experience and evidence from China. As a philanthropic phenomenon with Chinese characteristics, celebrity impression management reflects the uniqueness of China’s political, institutional, and social environment in influencing celebrity philanthropy and can provide some insights into the understanding of celebrities and philanthropy in other cultural contexts.

Why is this the case? Celebrity is “essentially a media production” (Giles, 2000). According to Boorstin’s argument, heroes create themselves, while celebrities are created by the media (Boorstin, 1992). Media coverage, especially interviews, is the most common way for celebrities to present themselves, and they often use media coverage in words, pictures, and videos to shape and present their image to the public. As forms of positive altruism, participation in philanthropic activities and support for non-profit programs are often used by Chinese celebrities for impression management.

We find that each interview is a well-designed and staged drama with active media attention, engaged fans, and retweets and comments from the public. To successfully demonstrate the social responsibilities that they portray and undertake, celebrities are accustomed to presenting a front-page image when interviewed by the media as a means of self-presentation (Rui and Stefanone, 2013). They adopt a variety of image management strategies to self-present their philanthropic motives (Baumeister and Hutton, 1987), and sometimes several strategies coexist. The presentation of celebrities’ philanthropic motives in media reports involves a process of self-impression management to control and guide the impressions of their philanthropic motives so that their philanthropic motives are “performative,” and their philanthropic images are self-constructed (Oyserman et al., 2012). In this process, the media play an indispensable supporting role.

As China’s media industrialization continues, the number of media outlets has increased dramatically, competition for readers has intensified, and the need for good news material has become increasingly urgent for all media outlets. Therefore, the media are very willing to cooperate and even incite celebrities to create news for the purpose of attracting public attention.

Celebrity-supported philanthropy is driven by the media’s thirst for stories and the publicity-grabbing imperatives of the celebrity industry (Jeffreys and Allatson, 2015). As a result, celebrity endorsement of not-for-profits is often held up as a major example of the perceived inauthentic nature of contemporary philanthropy (Batson et al., 1989).

Why do Chinese celebrities use impression management strategies for their charitable motives in the media? We argue that there are two main factors. First and foremost, celebrities in mainland China are not big citizens who act freely, as in Western countries. They often support charities under the encouragement and supervision of the government. Rather than exposing the self-centered egoism of China’s big citizens, the nature of this scrutiny underscores the politicized nature of wealth, fame and philanthropy in the Chinese context (Jeffreys, 2018). One of their main intentions in giving back to society is to receive government awards to justify their causes. As a legal framework of national charity awards and tax incentives, the “China Charity Award” provides a good policy environment for celebrities (Deng and Jeffreys, 2019).

Another important reason is that philanthropy has been conceptualized by celebrities as the consumption of cultural capital and, in some cases, interpreted as a merely expedient way to gain social capital or greater attention from fans (Anderson, 2009). A majority of celebrities are increasingly using media to mobilize their audiences for charitable and activist causes. In the media environment, fans have easier access, greater engagement, and stronger identification with celebrities’ philanthropic activism (Click et al., 2017), becoming an impetus for many celebrities to use the media as a tool to promote their charitable activities, form a strong social network and ensure a positive response from their fan networks.

Finally, we discuss the impact of China’s fragile social trust environment on the charitable image constructed by celebrities. Before the reform and opening up, there was no private wealth in China, but now that there is private wealth, some of the newly rich are ready to voluntarily donate some of their surplus funds (Mackey, 2005). Public distrust of the rich and famous has created problems for the development of a philanthropic culture in reform-era China (Jeffreys, 2011). Ordinary people tend to believe that those who have become newly rich through partial privatization are immoral because their wealth was acquired through corruption or theft of formerly publicly owned assets. Celebrities that acquire large amounts of wealth can easily attract criticism because the Chinese generally believe that individualistic wealth conflicts with the collective values of socialist collectivism. Socialist collectivism is vulnerable to criticism compared to model citizens in the era of production (Edwards and Jeffreys, 2010). In times of national disaster, entertainment celebrities are vulnerable to public censures if they do not make donations or if the amount of their donations is too low. Prompted by the public’s moral abduction of celebrities, to manage their social reputation, celebrities tend to gain fan support and public goodwill through self-constructed philanthropic motives.

## Conclusion

China’s philanthropy is entering a period of rapid development, but the overall transparency of the charity industry is not promising. The future of celebrity philanthropy depends not only on the country’s political and institutional environment but also on the availability of a trusting social environment. In the long run, China must further build a healthy public charity ecosystem, actively promote modern charity concepts, and enhance the credibility of the entire charity sector. In this way, the charitable behavior of celebrities can spread in an ethical manner and promote public prosociality. Moreover, promoting the healthy development of philanthropy requires the voluntary and active efforts of celebrities, rather than currying favor within a political framework. On the one hand, the media should portray and use celebrities’ charitable behavior more realistically to create news, improve the credibility of celebrities’ charitable images, and reduce the public’s unfounded speculation and questions about their charitable behavior. On the other hand, fans and the public must view celebrities’ charitable behavior in a more rational and professional manner. After all, there are individual differences in people’s motives for doing good deeds, and we must consider the possibility of different motives and trust those who perform good deeds sincerely.

As with any social science research, there are two limitations to this study that indicate the way for future research in this area. First, although this study found that the philanthropic motivations of celebrities presented in the media are the results of impression management, media culture might be a potential influencing factor. Celebrity is a production of the self specifically dependent upon a very elaborate and powerful media culture. For example, the media might intentionally exaggerate untruthful interviews (Marshall, 2010). Therefore, future research must consider media culture as a moderating factor. Second, although this study is illuminating from a theoretical perspective, it lacks empirical contributions, primarily because celebrities are difficult to recruit as research subjects. The data for the discourse analysis in this study came from public interviews in the media. Therefore, the findings cannot be simply and directly equated with celebrities’ true philanthropic motives but rather with their self-presenting motives. As data accessibility becomes more convenient and possible, future research must explore celebrities’ real philanthropic motivations to inform the fundraising actions of charitable organizations.

## Data Availability Statement

The original contributions presented in the study are included in the article/supplementary material, further inquiries can be directed to the corresponding author/s.

## Author Contributions

JG conceptualized, designed the study, and performed the analysis. JG and PZ wrote the first draft. PZ supervised the research. Both of the authors have read and agreed to the published version of the manuscript.

## Conflict of Interest

The authors declare that the research was conducted in the absence of any commercial or financial relationships that could be construed as a potential conflict of interest.

## Publisher’s Note

All claims expressed in this article are solely those of the authors and do not necessarily represent those of their affiliated organizations, or those of the publisher, the editors and the reviewers. Any product that may be evaluated in this article, or claim that may be made by its manufacturer, is not guaranteed or endorsed by the publisher.

## References

[B1] Anderson L. (2009). “Conspicuous charity,” in *Co-opting Culture: Culture and Power in Sociology and Cultural Studies*, eds Harden G. B. Carley R. (Lanham, MD: Lexington Books), 273.

[B2] Andreoni J. Petrie R. (2004). Public goods experiments without confidentiality: a glimpse into fund-raising. *J. Public Econ.* 88 1605–1623. 10.1016/S0047-2727(03)00040-9

[B3] Andreoni J. Gale W. G. Scholz J. K. Straub J. (1996). *Charitable contributions of time and money: University of Wisconsin–Madison Working Paper.* Madison, WI: University of Wisconsin.

[B4] Ariely D. Bracha A. Meier S. (2009). Doing good or doing well? Image motivation and monetary incentives in behaving prosocially. *Am. Econ. Rev.* 99 544–555. 10.1257/aer.99.1.544

[B5] Arkin R. M. (1981). Self-presentation styles. *Impression Manag. Theory Soc. Psychol. Res.* 311:334. 10.1016/B978-0-12-685180-9.50020-8

[B6] Basil D. Z. Ridgway N. M. Basil M. D. (2008). Guilt and giving: a process model of empathy and efficacy. *Psychol. Mark.* 25 1–23. 10.1002/mar.20200

[B7] Bator R. Cialdini R. (2000). The application of persuasion theory to the development of effective proenvironmental public service announcements. *J. Soc. Issues* 56 527–542. 10.1111/0022-4537.00182

[B8] Batson C. D. (1997). Self–other merging and the empathy–altruism hypothesis: reply to Neuberg et al. (1997). *J. Pers. Soc. Psychol.* 73 517–522. 10.1037/0022-3514.73.3.517

[B9] Batson C. D. Batson J. G. Griffitt C. A. Barrientos S. Brandt J. R. Sprengelmeyer P. (1989). Negative-state relief and the empathy—altruism hypothesis. *J. Pers. Soc. Psychol.* 56:922. 10.1037/0022-3514.56.6.922

[B10] Batson C. D. Lishner D. A. Stocks E. L. (2015). “The empathy—altruism hypothesis,” in *The Oxford Handbook Of Prosocial Behavior*, eds Schroeder D. A. Graziano W. G. (Oxford: Oxford University Press), 259–281.

[B11] Baumeister R. F. Hutton D. G. (1987). “Self-presentation theory: self-construction and audience pleasing,” in *Theories Of Group Behavior*, eds Mullen B. Goethals G. R. (New York, NY: Springer), 71–87. 10.1007/978-1-4612-4634-3_4

[B12] Boorstin D. J. (1992). *The Image: A Guide To Pseudo-Events In America.* New York, NY: Vintage.

[B13] Breeze B. Morgan G. G. (eds) (2009). “Philanthropy in a recession: an analysis of UK media representations and implications for charitable giving,” in *Proceedings Of The NCVO/VSSN Researching the Voluntary Sector Conference*, (Warwick: University of Warwick).

[B14] Brockington D. Henson S. (2015). Signifying the public: celebrity advocacy and post-democratic politics. *Int. J. Cult. Stud.* 18 431–448. 10.1177/1367877914528532

[B15] Charmaz K. (2014). *Constructing Grounded Theory.* Thousand Oaks, CA: Sage.

[B16] Cialdini R. B. Brown S. L. Lewis B. P. Luce C. Neuberg S. L. (1997). Reinterpreting the empathy–altruism relationship: when one into one equals oneness. *J. Pers. Soc. Psychol.* 73:481. 10.1037/0022-3514.73.3.481 9294898

[B17] Cialdini R. B. Schaller M. Houlihan D. Arps K. Fultz J. Beaman A. L. (1987). Empathy-based helping: is it selflessly or selfishly motivated? *J. Pers. Soc. Psychol.* 52:749. 10.1037/0022-3514.52.4.749 3572736

[B18] Click M. A. Lee H. Holladay H. W. (2017). ‘You’re born to be brave’: lady Gaga’s use of social media to inspire fans’ political awareness. *Int. J. Cult. Stud.* 20 603–619. 10.1177/1367877915595893

[B19] Dawson S. (1988). Four motivations for charitable giving: implications for marketing strategy to attract monetary donations for medical research. *J. Health Care Mark.* 8 31–37. 10287903

[B20] Deng G. Jeffreys E. (2019). Celebrity philanthropy in China: reconfiguring government and non-government roles in national development. *China Q.* 237 217–240. 10.1017/s0305741018001364

[B21] Drost E. A. (2011). Validity and reliability in social science research. *Educ. Res. Perspect.* 38 105–123.

[B22] Duygu G. Alison E. Deborah F. L. (2019). The effects of self-presentation to engage in physical activity. *Int. J. Exerc. Sci.* 12:263. 30899352 10.70252/PYCR4385 PMC6413838

[B23] Edwards L. Jeffreys E. (2010). *Celebrity In China.* Hong Kong: Hong Kong University Press. 10.5790/hongkong/9789622090873.001.0001

[B24] Fraser B. P. Brown W. J. (2002). Media, celebrities, and social influence: identification with Elvis Presley. *Mass Commun. Soc.* 5 183–206. 10.1207/S15327825MCS0502_5 33486653

[B25] Fultz J. Batson C. D. Fortenbach V. A. McCarthy P. M. Varney L. L. (1986). Social evaluation and the empathy–altruism hypothesis. *J. Pers. Soc. Psychol.* 50:761. 10.1037/0022-3514.50.4.761 3712222

[B26] Fultz J. Schaller M. Cialdini R. B. (1988). Empathy, sadness, and distress three related but distinct vicarious affective responses to another’s suffering. *Pers. Soc. Psychol. Bull.* 14 312–325. 10.1177/014616728801400201 30045481

[B27] Giles D. (2000). *Illusions Immortality: A Psychology Of Fame And Celebrity.* London: Macmillan International Higher Education. 10.1007/978-1-137-09650-0

[B28] Glazer A. Konrad K. A. (1996). A signaling explanation for charity. *Am. Econ. Rev.* 86 1019–1028.

[B29] Goffman E. (1949). Presentation of self in everyday life. *Am. J. Sociol.* 55 6–7.

[B30] Guo Y. Ren W. (2020). Managing image: the self-praise of celebrities on social media. *Discourse Context Media.* 38:100433. 10.1016/j.dcm.2020.100433

[B31] Guy B. S. Patton W. E. (1989). The marketing of altruistic causes: understanding whypeople help. *J. Consum. Mark.* 6 19–30.

[B32] Harbaugh W. T. (1998). The prestige motive for making charitable transfers. *Am. Econ. Rev.* 88 277–282.

[B33] Hassid J. Jeffreys E. (2015). Doing good or doing nothing? Celebrity, media and philanthropy in China. *Third World Q.* 36 75–93. 10.1080/01436597.2015.976019

[B34] Hibbert S. Horne S. (1996). Giving to charity: questioning the donor decision process. *J. Consum. Mark.* 13 4–13.

[B35] Hodges B. D. Kuper A. Reeves S. (2008). Discourse analysis. *BMJ* 337:a879. 10.1108/07363769610115366 18687729

[B36] Jackson T. D. (2001). Young african americans: a new generation of giving behaviour. *Int. J. Nonprofit Volunt. Sect. Mark.* 6 243–253. 10.1002/nvsm.150

[B37] Jeffreys E. (2011). Zhang ziyi and china’s celebrity-philanthropy scandals. *Portal* 8 1–21. 10.5130/portal.v8i1.1627

[B38] Jeffreys E. (2015). Celebrity philanthropy in mainland China. *Asian Stud. Rev.* 39 571–588. 10.1080/10357823.2015.1081871

[B39] Jeffreys E. (2018). “Celebrity philanthropy in China: rethinking cultural studies’‘big citizen’critique,” in *Routledge Handbook of Celebrity Studies*, ed. Elliot A. (Abingdon: Routledge), 227–242. 10.4324/9781315776774-15

[B40] Jeffreys E. Allatson P. (2015). “Celebrity philanthropy: an introduction,” in *Celebrity Philanthropy*, eds Jeffreys E. Allatson P. (Chicago, IL: The University of Chicago Press), 1–16. 10.4324/9781315306872-1

[B41] Jørgensen M. W. Phillips L. J. (2002). *Discourse Analysis As Theory And Method.* Thousand Oaks, CA: Sage. 10.4135/9781849208871

[B42] Khalifeh Soltani M. Goudarzi M. Dehghan Ghahfarokhi A. Alidoust Gahfarokhi E. (2021). Presenting the model of social responsibility for sports celebrities. *Sci. J. Organ. Behav. Manag. Sport Stud.* 8 41–50.

[B43] Landis J. R. Koch G. G. (1977). The measurement of observer agreement for categorical data. *Biometrics* 33 159–174. 10.2307/2529310 843571

[B44] Levy K. Pissler K. B. (2020). *Charity With Chinese Characteristics: Chinese Charitable Foundations Between the Party-state and Society.* Cheltenham: Edward Elgar Publishing. 10.4337/9781788115070

[B45] Lwin M. Phau I. (2014). An exploratory study of existential guilt appeals in charitable advertisements. *J. Mark. Manag.* 30 1467–1485. 10.1080/0267257x.2014.939215

[B46] Mackey M. (2005). *The New Chinese Philanthropy Asia Times.* Available Online At: http://www.atimes.com/atimes/China/GE14Ad06.html [accessed May 14, 2005]

[B47] Mannino C. A. Snyder M. Omoto A. M. (2011). “Why do people get involved? Motivations for volunteerism and other forms of social action,” in *Social Motivation*, ed. Dunning D. (Hove: Psychology Press), 127–146.

[B48] Marshall P. D. (2010). The promotion and presentation of the self: celebrity as marker of presentational media. *Celebr. Stud.* 1 35–48. 10.1080/19392390903519057

[B49] Marwick A. Boyd D. (2011). To see and be seen: celebrity practice on Twitter. *Convergence* 17 139–158. 10.1177/1354856510394539

[B50] McDonald N. Schoenebeck S. Forte A. (2019). Reliability and inter-rater reliability in qualitative research: norms and guidelines for CSCW and HCI practice. *Proc. ACM Hum. Comput. Interact.* 3 1–23. 10.1145/335917434322658

[B51] Merchant A. Ford J. B. Sargeant A. (2010). Charitable organizations’ storytelling influence on donors’ emotions and intentions. *J. Bus. Res.* 63 754–762. 10.1016/j.jbusres.2009.05.013

[B52] Ostrander S. A. (1997). Why the wealthy give: the culture of elite philanthropy. *Contemp. Sociol.* 26:160. 10.2307/2076753

[B53] Oyserman D. Elmore K. Smith G. (2012). “Self, self-concept, and identity,” in *Handbook of Self And Identity*, eds Leary M. R. Tangney J. P. (New York, NY: The Guilford Press), 69–104.

[B54] Palmer C. (2021). Charity, social justice and sporting celebrity foundations. *Celebr. Stud.* 12 565–580. 10.1080/19392397.2019.1691029

[B55] Paulhus D. L. Trapnell P. D. (2008). “Self-presentation of personality: an agency-communion framework,” in *Handbook of Personality: Theory and Research* eds John O. P. Robins R. W. Pervin L. A. (New York: The Guilford Press), 492–517.

[B56] Pharoah C. Tanner S. (1997). Trends in charitable giving. *Fisc. Stud.* 18 427–443. 10.1111/j.1475-5890.1997.tb00272.x

[B57] Potter J. (2003). “Discourse analysis and discursive psychology,” in *Qualitative Research In Psychology: Expanding Perspectives In Methodology And Design*, eds Camic P. M. Rhodes J. E. Yardley L. (Washington, DC: American Psychological Association), 73–94. 10.1037/10595-005

[B58] Potter J. Wetherell M. (1987). Discourse analysis. *Routledge Handb. Discourse Anal.* 104 1125–1149.

[B59] Ramsey G. Brandon R. (2011). Why reciprocal altruism is not a kind of group selection. *Biol. Philos.* 26 385–400. 10.1007/s10539-011-9261-7

[B60] Reinstein D. Riener G. (2012). Reputation and influence in charitable giving: an experiment. *Theory Decis.* 72 221–243. 10.1007/s11238-011-9245-8

[B61] Rui J. Stefanone M. A. (2013). Strategic self-presentation online: a cross-cultural study. *Comput. Hum. Behav.* 29 110–118. 10.1016/j.chb.2012.07.022

[B62] Schlegelmilch B. B. Diamantopoulos A. Love A. (1997). Characteristics affecting charitable donations: empirical evidence from Britain. *J. Mark. Pract.* 3 14–28. 10.1108/eum0000000004318

[B63] Sokolowski S. W. (1996). Show me the way to the next worthy deed: Towards a microstructural theory of volunteering and giving. *Voluntas* 7 259–278. 10.1007/bf02354118

[B64] Steele W. R. Schreiber G. B. Guiltinan A. Nass C. Glynn S. A. Wright D. J. (2008). The role of altruistic behavior, empathetic concern, and social responsibility motivation in blood donation behavior. *Transfusion* 48 43–54. 10.1111/j.1537-2995.2007.01481.x 17894795

[B65] Tainsky S. Babiak K. (2011). Professional athletes and charitable foundations: an exploratory investigation. *Int. J. Sport Manag. Mark.* 9 133–153. 10.1504/ijsmm.2011.041568

[B66] Valentinsson M.-C. (2018). Stance and the construction of authentic celebrity persona. *Lang. Soc.* 47 715–740. 10.1017/S0047404518001100

[B67] Van den Bulck H. (2018). *Celebrity Philanthropy And Activism: Mediated Interventions In The Global Public Sphere.* Abingdon: Routledge. 10.4324/9781315306872

[B68] Wu D. Lin M. (2017). “Speech acts and facework by Chinese celebrities on Weibo,” in *Politeness Phenomena Across Chinese Genres*, ed. Chen X. (Sheffield: Equinox), 119–134.

[B69] Zhang M. Wu D. D. (2018). A cross-cultural analysis of celebrity practice in microblogging. *East Asian Pragmat.* 3 179–200. 10.1558/eap.33060

